# Evaluation of the Effect of Transcatheter Aortic Valve Implantation in Patients with Severe Aortic Stenosis on the Concentration of the Fatty Acids Involved in Inflammation

**DOI:** 10.3390/metabo15120774

**Published:** 2025-11-29

**Authors:** Tomasz Figatowski, Wiktoria Karos, Joanna Marlęga-Linert, Ludmiła Hasak, Agnieszka Kuchta, Gabriela Chyła-Danił, Agnieszka Ćwiklińska, Monika Czapiewska, Marcin Fijałkowski, Marcin Gruchała, Radosław Targoński, Dariusz Jagielak, Adriana Mika, Agnieszka Mickiewicz

**Affiliations:** 1 First Department of Cardiology, Medical University of Gdansk, Debinki 1, 80-211 Gdansk, Poland; wiktoriakaros@gumed.edu.pl (W.K.); asia.marlega@gmail.com (J.M.-L.); marcin.fijalkowski@gumed.edu.pl (M.F.); mgruch@gumed.edu.pl (M.G.); rtarg@gumed.edu.pl (R.T.); amickiewicz@gumed.edu.pl (A.M.); 2 University Clinical Centre, Smoluchowskiego 17, 80-952 Gdansk, Poland; dariusz.jagielak@gumed.edu.pl; 3 Department of Anaesthesiology and Intensive Care, Medical University of Gdansk, Debinki 1, 80-211 Gdansk, Poland; luudmilahasak@gmail.com; 4 Department of Clinical Chemistry, Medical University of Gdansk, Debinki 1, 80-211 Gdansk, Poland; agakuchta@gumed.edu.pl (A.K.); gabriela.chyla-danil@gumed.edu.pl (G.C.-D.); agnieszka.cwiklinska@gumed.edu.pl (A.Ć.); 5 Department of Pharmaceutical Biochemistry, Faculty of Pharmacy, Medical University of Gdansk, Debinki 1, 80-211 Gdansk, Poland; monika.czapiewska99@gumed.edu.pl; 6 Department of Cardiac Surgery, Medical University of Gdansk, Debinki 1, 80-211 Gdansk, Poland; 7 Department of Environmental Analysis, Faculty of Chemistry, University of Gdansk, Wita Stwosza 63, 80-308 Gdansk, Poland

**Keywords:** polyunsaturated fatty acids, monounsaturated fatty acids, eicosapentaenoic acid, docosahexaenoic acid, chronic inflammation, cardiovascular disease, aortic stenosis, transcatheter aortic valve implantation, fatty acids, biomarkers

## Abstract

Background: Pathogenesis of aortic stenosis (AS) involves lipid infiltration, inflammation, and oxidative stress, which drive calcification of the aortic valve and progression to heart failure (HF). Fatty acids (FAs) play a crucial role in these processes. A treatment option for severe symptomatic AS in elderly and high-risk patients is transcatheter aortic valve implantation (TAVI). Objective: To investigate the change in FA profiles in patients undergoing TAVI. Methods: This single-center prospective study included 25 patients with severe AS qualified for TAVI procedure. Blood samples were collected before TAVI and after six months. FA profiles were analyzed by gas chromatography-electron ionization mass spectrometry. Results: Notable changes were identified in FA profiles, including a reduction in docosahexaenoic acid (DHA) levels (117 ± 48.0 µM vs. 141 ± 53.0 µM, *p* = 0.001) and an increase in alpha-linolenic acid (ALA) concentration (32.8 ± 12.3 µM vs. 19.9 ± 6.40 µM, *p* = 0.003) six months post-TAVI. Additionally, significant elevations were noted in specific medium-chain FAs (C12) and branched-chain fatty acids (iso C16, iso C17 and anteiso C15, anteiso C17) at six months after TAVI. However, total n-3 polyunsaturated fatty acids (n3 PUFA) levels decreased (*p* = 0.039), while n-6 polyunsaturated fatty acids (n6 PUFA) levels exhibited no significant overall change at this time point. Decrease in mean pressure gradient (PG) was negatively correlated with eicosapentaenoic acid (EPA), DHA, n-3 docosapentaenoic acid (DPA n3) and n3 PUFA levels in a six-month observation. Conclusions: Our results underscore the complex interplay between cardiac intervention and FA changes, providing novel insights into the metabolic impact of TAVI on FAs serum profile.

## 1. Introduction

Aortic stenosis (AS) is the most prevalent heart valve disease, with increasing occurrence alongside the aging population. Significant narrowing of the aortic valve area (AVA) leads to increased afterload, left ventricular hypertrophy, adverse remodeling and further development of heart failure (HF) with reduced left ventricular ejection fraction (LVEF) [1].

The pathogenesis of severe calcification in the aortic valve includes lipid infiltration, inflammation and oxidative stress, all of which collectively foster further calcification.

The key step in the pathogenesis of fibrocalcific remodeling in AS is inflammation driven by lipids. Inflammatory mediators, cytokines and tumor necrosis factors, cause imbalance of synthesis and degradation of extracellular matrix (ECM). The ECM malfunction plays a role in the initial stage of aortic leaflets calcification.

The initial stage involves mechanical stress and endothelial injury, especially on the aortic side of the valve leaflets. This leads to infiltration of low-density lipoprotein (LDL) and lipoprotein(a) [Lp(a)] into the subendothelial space. These lipoproteins undergo oxidation, triggering local inflammation [2]. Oxidized LDL and other pro-inflammatory stimuli attract macrophages and T lymphocytes, which release cytokines (e.g., TNF-α, Interleukine-1β, Interleukine-6) and matrix metalloproteinases (MMPs). These factors contribute to valve interstitial cell (VIC) activation and matrix remodeling [3].

Inflammatory mediators drive the expression of osteogenic genes, like bone morphogenic protein 2 (BMP2) in valvular interstitial cells.

VICs, under the influence of inflammatory signals and mechanical stress, differentiate into osteoblast-like cells. This process is driven by bone morphogenetic proteins (BMPs), especially BMP-2 and the Wnt/β-catenin pathway, leading to the expression of osteogenic markers such as RUNX2 and osteocalcin [4]. This results in the deposition of hydroxyapatite crystals within the valve tissue, leading to the production of calcified nodules that progressively stiffen the valve leaflets.

High dietary intake of even-chained saturated fatty acids (ECSFAs) and total saturated fatty acids (SFAs) contributes to calcific aortic valve disease (CAVD), mainly by increasing the concentration of atherogenic lipoproteins, with the greatest impact on low-density lipoprotein cholesterol (LDL-C) [5]. The development of AS is associated with marked changes in aortic valve FAs. It has been shown that the aortic valve contains high amounts of saturated palmitic acid (c16:0) and omega-9 oleic acid (C18:1n9).

Another FAs subgroup, branched-chain fatty acids (BCFAs), decrease the total cholesterol (TC) level, which may contribute to the reduction in atherosclerotic cardiovascular disease (ASCVD) and AS risk [6]. Pentadecanoic acid (15:0) and heptadecanoic acid (17:0) belonging to the odd-chained fatty acids (OCFAs) show an inverse correlation with cardiovascular disease (CVD) risk, while inflammation and oxidative stress are positively associated [7]. The n6 polyunsaturated fatty acids (PUFAs), such as arachidonic acid (ARA), linoleic acid (LA), are substrates for the synthesis of signaling molecules that tend to trigger a pro-inflammatory response. Elevated levels of ARA lead to an increase in the production of pro-inflammatory eicosanoids, potentially accelerating atherosclerosis progression and aortic valve calcification [8,9]. In contrast, the n3 PUFAs, docosahexaenoic acid (DHA) and eicosapentaenoic acid (EPA), cause an inflammation-resolving effect through n3 PUFA-derived mediators. These n3 PUFAs have a favorable impact on aortic valve calcification by blocking the entry of inflammatory cells, aiding in the clearance of apoptotic cells, and promoting the removal of immune cells [8,10]. Monounsaturated fatty acids (MUFAs) exert anti-inflammatory proprieties, which may be favorable in the context of inhibition of AS progression. However, the specific impact of different MUFA representatives on AS requires further investigation [11].

**Figure 1:**
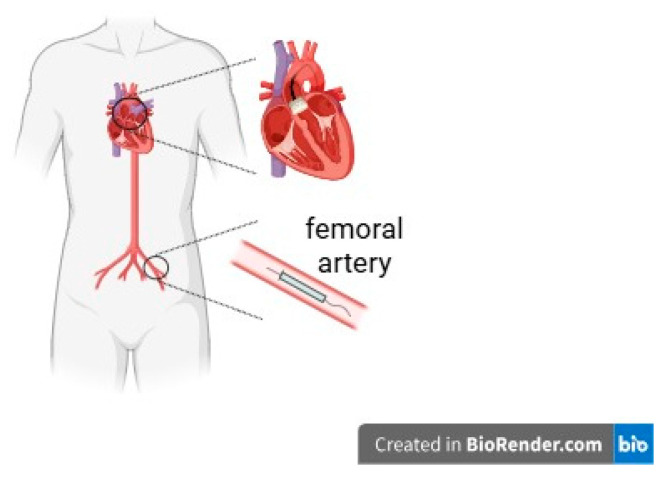
Procedure of transcatheter aortic valve implantation TAVI in aortic stenosis.

In untreated symptomatic severe AS, the annual risk of death is approximately 25%, although it varies depending on the AS subtype [12]. Currently, there is no pharmacological treatment available for severe AS or the concomitant HF; the only treatment option remains aortic valve replacement (AVR) [13]. A rapidly developing minimally invasive treatment method for severe AS is transcatheter aortic valve implantation (TAVI) (Figure 1). TAVI has become more widespread and is now the preferred treatment option for elderly patients with severe symptomatic AS, regardless of whether they are at high, moderate or even low surgical risk. Compared to surgical aortic valve replacement (SAVR), TAVI offers advantages like minimized hospital stay durations, enhanced recovery efficiency and prompt resumption of routine activities. TAVI significantly improves hemodynamic parameters, including reducing the transvalvular gradient below 20 mmHg and increasing the valve orifice area to 1.5–2.0 cm [14]. According to CoreValve studies, the one-year survival rate for high-risk patients with severe AS undergoing TAVI was higher than for those who had undergone SAVR [15]. The 5-year follow-up of this cohort demonstrated comparable mid-term survival rates between the two methods [16].

The specific pathogenetic aspects of AS and their impact on the effectiveness of TAVI are still not fully understood. There are limited data on the role of FAs in the pathogenesis and progression of AS and even fewer studies that explore their changes after TAVI [17,18]. The aim of our study was to analyze the change in FA profiles in patients undergoing TAVI.

## 2. Materials and Methods

This single-center prospective study was conducted at the First Department of Cardiology of the Medical University of Gdansk, Poland. We included 25 patients with severe aortic stenosis who were qualified for the TAVI procedure as a routine standard of care. The study was conducted in accordance with the Declaration of Helsinki. The study protocol was approved by the Local Bioethics Committee at the Medical University of Gdansk. Informed consent was obtained from all patients (protocol no. KB/415/2025).

### 2.1. Patients

The study included 25 individuals diagnosed with severe, high gradient AS (12 men and 13 women). Blood samples were collected before the procedure, immediately after the procedure, and one day after the procedure from a central venous catheter (CVC) whose distal tip was positioned in the superior vena cava just above the entry to the right atrium. The additional sampling time points (immediately after and one day after the procedure) were established to assess oxidative stress parameters and to investigate potential changes in periprocedural biomarkers. However, preliminary results did not reveal any significant alterations. All blood samples at all time points were collected in a fasting state (minimum 10 h). Further, blood samples were processed into serum by centrifugation at 1000× *g* for 15 min and separated to Eppendorf (Medlab Products), then stored in −80 °C. ECHO measurements were performed 24–48 h after TAVI.

We assessed the severity of AS in routine echocardiography with parameters such as mean AV pressure gradient (AV mean PG), aortic valve velocity (AV max) and aortic valve area (AVA). The AV mean PG is a fundamental aortic valve hemodynamic parameter for assessing the severity and clinical significance of AS. Calculated primarily by Doppler echocardiography, it reflects the average systolic pressure difference between the left ventricle and aorta and correlates directly with the severity of obstruction of the blood flow through the aortic valve. Overall, in patients with AS the AV mean PG remains a key determinant in diagnosis, monitoring and management decisions, guiding timing for aortic valve intervention. Typical high gradient severe AS is associated with an AV mean PG above 40 mmHg.

Clinical characteristic of the study group, showing the number of patients with medical conditions is presented in Table 1. The mean age of the cohort was 81 ± 5.6 years. Most participants presented with overweight, arterial hypertension (HA), hypercholesterolemia and HF symptoms (Table 1). Coronary artery disease (CAD) and chronic kidney disease (CKD) were also prevalent within this population, reflecting a high burden of comorbidities commonly associated with advanced aortic stenosis. Selected biochemical and anthropometric characteristics in the study population are contained in Table 2.

Statins were the only class of medications influencing changes in lipid levels among the study population. Prior to TAVI, 96% of patients were receiving statin therapy, whereas only 56% continued treatment six months after the procedure.

### 2.2. TAVI Procedure

All patients underwent the TAVI procedure through femoral artery according to routine, standard protocol. Biological aortic valve prosthesis was implanted through a catheter and then implanted at the site of the stenotic native valve. The procedure was performed under fluoroscopic guidance. Indications for TAVI included patients over 75 years of age with severe symptomatic AS and intermediate or high surgical risk, or patients with contraindications to surgical aortic valve replacement (SAVR) [17].

### 2.3. Chemicals

Chloroform, dichloromethane and methanol (LC-MS grade), as well as n-hexane, potassium hydroxide, hydrochloric acid (36–38%), sodium chloride and physiological solution (pure for analysis grade) were purchased from POCH (Poland). A 10% boron trifluoride in methanol solution and standards of straight-chain fatty acids were purchased from Sigma-Aldrich (Germany). Standards of branched-chain fatty acids and very long-chain fatty acids were purchased from Larodan (Sweden).

### 2.4. Fatty Acids Analysis

Total lipids were extracted from serum using a chloroform–methanol mixture (2:1, *v*/*v*). The lipid extracts were dried by evaporation under a stream of nitrogen. The fatty acids are hydrolyzed from the total esterified lipids, but the samples also contained FA, which was present in the samples in unesterified form. Each sample was hydrolyzed with 1 mL of 0.5 M KOH in methanol at 90 °C for 3 h. The mixture was acidified with 0.2 mL of 6 M HCl and 1 mL of water was added. The non-esterified FAs were extracted three times with 1 mL n-hexane and evaporated to dryness under a stream of nitrogen. FA methyl esters (FAMEs) were prepared with 1 mL of a 10% boron trifluoride in methanol solution (90 min at 55 °C); 1 mL of water was added to the reaction mixture, the FAMEs were extracted three times with 1 mL of n-hexane and the solvent was evaporated.

FAMEs were analyzed by gas chromatography-electron ionization mass spectrometry QP-2020 NX (Shimadzu, Japan) and separated on a 30 m 0.25 mm i.d. Zebron ZB-5MSi capillary column (film thickness 0.25 mm). One μL of the sample was injected at a split mode (ratio 20:0). The temperature of injection, ion source and transfer line were 300 °C, 200 °C and 300 °C, respectively. The column temperature was set to a range of 60 to 300 °C (4 °C/min), with helium as the carrier gas at the column head pressure of 60 kPa. The electron energy used for FAME ionization was 70 eV; 19-methylarachidic acid was used as an internal standard. Full scan mode was used with a mass scan range of *m*/*z* 45 to 700. Accurate identification of the FA profile was possible due to the use of a reference.

### 2.5. Quantification Protocol and Statistical Analysis

After extraction, the mass of lipids was weighed, and based on the total volume of serum used for extraction, we calculated the amount of lipids per 1 mL of serum. In order to quantify the total number of FAs, 19-methylarachidic acid was used as an internal standard. Accurate identification of the FA profile was achieved using reference standards (BCFA and VLCFA standards, Larodan, Solna, Sweden, and 37 FAME Mix, Sigma-Aldrich). FA concentrations were calculated based on the internal standard signal and expressed as relative abundance. These values were then calculated for a specific serum volume, and then finally, the results are shown as a µmol of FA/1L of serum. Data analysis was performed using Sigma Plot 14.5 software (Systat Software Inc., San Jose, CA, USA). Results are presented as mean and standard deviation (SD). Statistical significance was set at *p* < 0.05. The figures were created in Excel Microsoft 365. The Shapiro–Wilk test was used to assess the normality of the data distribution. The paired Student *t*-test was used to assess differences between the two groups (before and 6 m after TAVI).

## 3. Results

### 3.1. Effect of TAVI on Lipid Parameters

**Figure 2:**
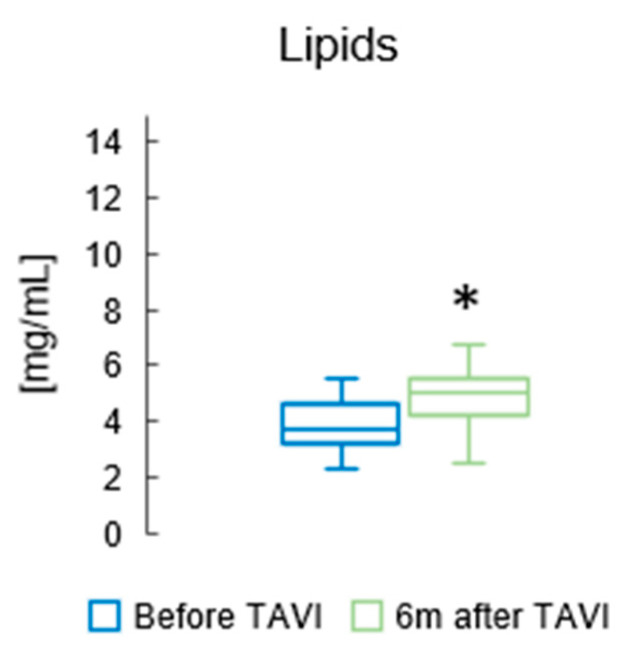
The total concentration of lipids in the serum of patients before and six months after TAVI. * *p* < 0.05 for paired Student *t*-test.

Figure 2 presents the total concentration of lipids in the serum of patients before and six months after TAVI. We found that the concentration of total lipids in patients’ serum was higher after TAVI, than before (*p* = 0.014; Figure 2).

Lipid parameters before TAVI and after six months are presented in Table 3. A significant increase in TC levels was observed six months following TAVI (*p* = 0.001). TG concentrations also demonstrated a marked elevation at the same time point (*p* < 0.001). Additionally, HDL-C levels showed a notable rise after the procedure (*p* = 0.005), and a similar trend was identified for LDL-C, with a substantial growth recorded at six months post-intervention (*p* = 0.010).

### 3.2. Effect of TAVI on the Concentration of Fatty Acids Involved in Inflammation

**Figure 3:**
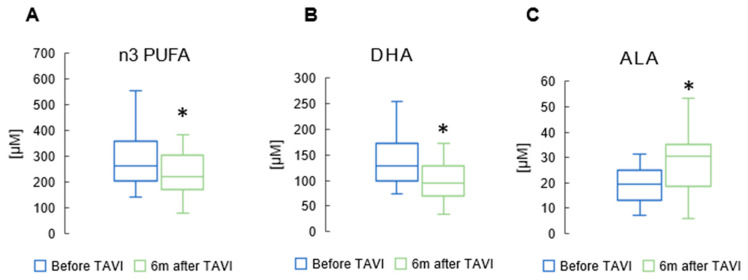
Concentration of total n3 polyunsaturated fatty acids (n3 PUFAs) (**A**); docosahexaenoic acid (DHA) (**B**); and alpha-linolenic acid (ALA) (**C**) in serum of patients after TAVI. * *p* < 0.05 for paired Student *t*-test.

Supplementary Table S1 shows patients’ serum FA profiles after the TAVI procedure. We found that among n3 PUFAs, DHA concentration was significantly reduced after six months (117 ± 48.0 µM vs. 141 ± 53.0 µM, *p* = 0.001) compared to baseline. The levels of ALA increased significantly six months after procedure compared to the baseline (32.8 ± 12.3 µM vs. 19.9 ± 6.40 µM, *p* = 0.003). However, we observed a substantial decrease in levels of all n3 PUFAs six months post-TAVI (265 ± 105 µM vs. 284 ± 112 µM, *p* = 0.039). (Figure 3).

**Figure 4:**
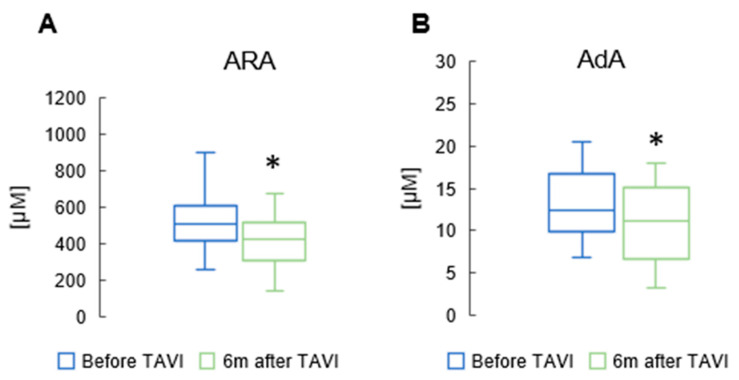
Change in concentrations of arachidonic acid (ARA) (**A**), and adrenic acid (AdA) (**B**) in the serum of patients 6 m after TAVI. * *p* < 0.05 for paired Student *t*-test.

In the n6 PUFAs group, there was a notable reduction in ARA and AdA levels six months after the procedure (462 ± 119 µM vs. 515 ± 154 µM, *p* = 0.015; 11.6 ± 4.48 µM vs. 12.3 ± 4.23 µM, *p* = 0.05, respectively) (Figure 4). However, there were no significant differences in the values of other n6 PUFAs before and six months after TAVI.

### 3.3. Effect of TAVI on the Concentration of Branched Chain and Monounsaturated Fatty Acids

**Figure 5:**
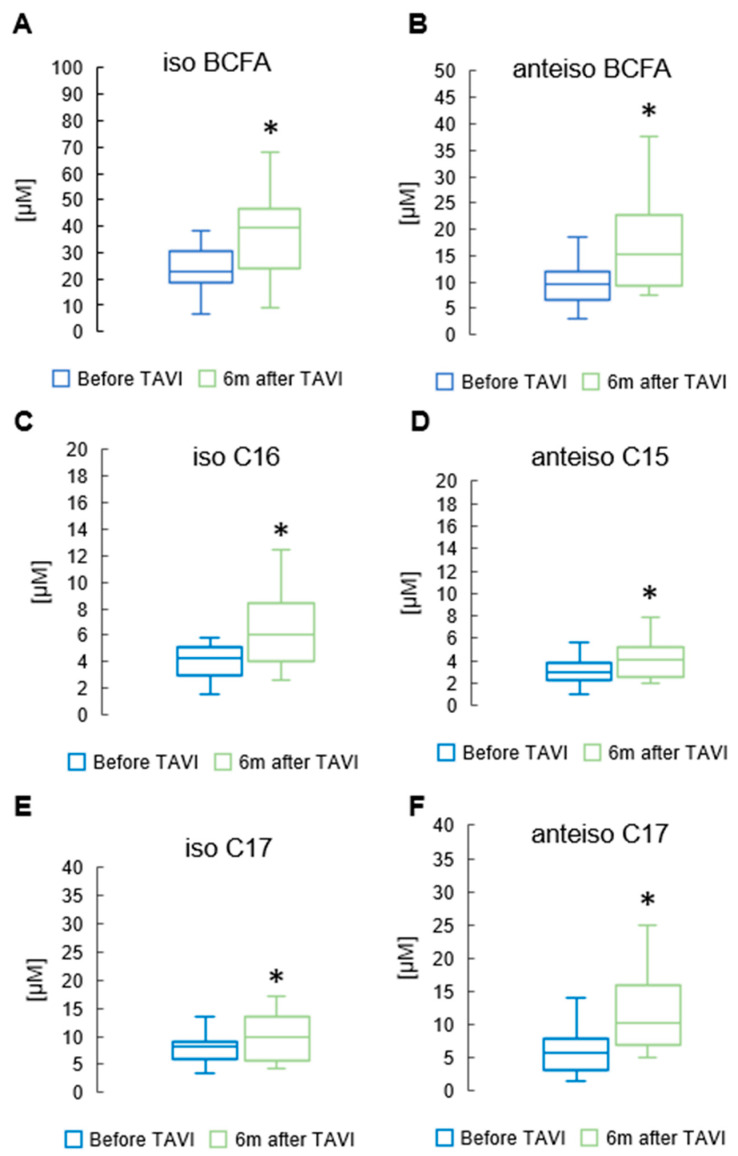
Change in concentrations of total and iso branched chain fatty acids (iso BCFA) (**A**); total anteiso BCFA (**B**); and selected BCFA (**C**–**F**) in the serum of patients 6 m after TAVI. * *p* < 0.05 for paired Student *t*-test.

**Figure 6:**
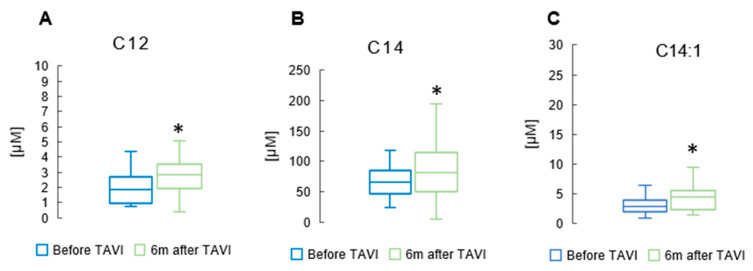
Change in concentrations of medium-chain FA lauric acid (C12) (**A**); long-chain FA myristic acid (C14) (**B**); and monounsaturated FA myristoleic acid (C14:1) (**C**) in the serum of patients 6 m after TAVI. * *p* < 0.05 for paired Student *t*-test.

After six months, analysis revealed a notable rise in the concentration of total BCFAs, total iso BCFA and total anteiso BCFA compared to pre-TAVI levels (*p* = 0.013, *p* = 0.001, *p* = 0.001, Respectively) (Figure 5) (Supplementary Table S1). In the iso BCFA group, the concentrations of isoC16 and isoC17 showed a significant increase after six months compared to pre-procedure levels (5.66 ± 2.17 µM vs. 4.51 ± 1.46 µM, *p* = 0.001 and 9.13 ± 3.81µM vs. 8.19 ± 2.92 µM, *p* = 0.045, respectively). Analysis revealed a significant rise in anteiso C17 values after six months compared to pre-procedure levels (10.2 ± 4.11 µM vs. 7.07 ± 3.27 µM, *p* = 0.001). Anteiso C15 showed a slight increase after the procedure (4.31 ± 2.06 µM vs. 3.02 ± 1.14 µM, *p* = 0.044) (Supplementary Table S2). Figure 6 displays significant increases in medium-chain FA measurements observed six months after the TAVI procedure compared to pre-procedure values: C12 (2.45 ± 1.23 µM vs. 1.84 ± 0.89 µM, *p* = 0.039). Apart from C14 and C14:1, we did not observe statistically significant changes among long-chain fatty acids (LCFAs) and monounsaturated fatty acids (MUFAs) (Supplementary Table S1).

### 3.4. Effect of TAVI on the Concentration of Odd and Even Chain Saturated Fatty Acids

Total OCFA levels did not significantly change between pre-TAVI and six months post-procedure (Supplementary Table S1). Also, there were no notable changes in total SFA and total ECSFA levels between the pre-TAVI period and six months after the procedure.

### 3.5. Aortic Valve Parameters and FA Concentrations

We observed significant correlations between aortic valve hemodynamic parameters and specific serum FA levels. Notably, a negative correlation was identified between the maximum pressure gradient (max PG) of the aortic valve and the concentrations of DHA (R = −0.488, *p* < 0.05), n3 PUFA (R = −0.569, *p* < 0.05) and iso C16:0 (R = −0.440 *p* < 0.05). A reduction in max PG over a 6-month period was associated with increased levels of DHA, n-3 PUFA and iso C16:0. Similarly, a negative correlation was detected between the mean pressure gradient (mean PG) and the concentrations of EPA (R = −0.452, *p* < 0.05), DHA (R = −0.543, *p* < 0.05), DPA n3 (R = −0.486, *p* < 0.05) and n3 PUFA (R = −0.633, *p* < 0.05). Longitudinal analysis revealed that a decrease in mean PG was paralleled by an increase in EPA, DHA, DPA n3 and n3 PUFA levels after six months. Conversely, we found a positive correlation between maximum aortic valve velocity (Vmax) and levels of LA (R = 0.436, *p* < 0.05), total n6 PUFA (R = 0.409, *p* < 0,05), iso C15:0 (R = 0.458, *p* < 0,05), iso C17:0 (R = 0.490, *p* < 0,05), anteiso C15:0 (R = 0.601, *p* < 0.05), anteiso C17:0 (R = 0.458, *p* < 0.05), total BCFA (R = 0.434, *p* < 0,05) and MUFA (R = 0.427, *p* < 0,05). Over the 6-month follow-up, a decrease in Vmax was accompanied by a decline in the concentrations of these FAs species. Over the 6-month period, we did not observe a significant correlation between SFA concentrations and aortic valve parameters.

We also measured FA concentrations immediately after TAVI and on the day following the procedure. Statistically significant changes in the studied patients, apart from those observed six months after the procedure, were found in the concentration of C18 (after TAVI, the concentration was lower than before the procedure); C28 and C25 (the day after the procedure, FA concentrations were higher than before TAVI); DPA n3 (the concentration was lower immediately after TAVI compared to before the procedure); and EDA (the concentration was lower the day after TAVI compared to before TAVI) (Supplementary Table S2).

## 4. Discussion

This study evaluated the effect of TAVI on serum lipid and FA concentrations over a 6-month period. Our findings indicate notable changes in FA profiles: (1) reduction in docosahexaenoic acid concentration after TAVI; (2) an increase in alpha-linolenic acid concentration six months post-TAVI; (3) rise in specific medium-chain FAs (C12) and anti-inflammatory BCFAs at six months; and (4) decrease in pro-inflammatory ARA and anti-inflammatory AdA. These results may suggest that improved hemodynamics after TAVI influence metabolism of fatty acids particularly those involving PUFAs and BCFAs. Notably, reductions in pro-inflammatory ARA and increases in anti-inflammatory BCFAs highlight the post-procedure metabolic adaptation. We found also significant correlations between improved AV max/mean gradient, Vmax and specific serum FA levels indicating a potential link between FAs metabolism and valvular function. Decrease in mean PG was paralleled by an increase in EPA, DHA, DPA n3 and n3 PUFA levels in a six-month observation.

Previously, a study of a group of patients with bicuspid aortic valve stenosis who underwent TAVI revealed that more severe baseline hemodynamic impairment and suboptimal left venctricle (LV) recovery (poor LV reverse remodeling) after TAVI were associated with increased activity in the ARA metabolism pathway, as evidenced by significantly elevated levels of ARA and its metabolites, including 15-ketoeicosatetraenoic acid (15-KETE), 15-hydroxyeicosatetraenoic acid (15(S)-HETE), prostaglandin G2, thromboxane B2, leukotriene A4 and leukotriene B4 [19].

Our study showed notable reduction in ARA levels six months after TAVI in patients with tricuspid AS. Given its pivotal role in inflammation, mitigating the ARA pathway may be critical for halting the pathological progression of aortic valve degeneration.

Several studies have demonstrated that n3 PUFAs provide beneficial effects by reducing aortic valve thickening and calcification, thereby slowing the progression of aortic valve disease. In human stenotic aortic valves, n3 PUFAs were more abundantly deposited in noncalcified areas compared with calcified areas. All patients included in our study exhibited severely degenerated and extensively calcified aortic valves [11]. Thus, the observed changes in n3 PUFA levels appear to be a consequence of TAVI, which entails resecting calcified regions from the aortic orifice. We found a significant decrease in total n3 PUFA and DHA concentrations six months after TAVI. However, at six months post-TAVI, levels of certain n3 PUFAs (ALA, EPA and ETA) were significantly elevated, potentially imparting long-term anti-inflammatory effects that may protect the prosthesis from deterioration. An increase in ALA concentrations six months after TAVI may represent the improved aortic valve status, as previous studies showed that ALA was inversely associated with aortic valve calcium (AVC), a predecessor of aortic valve stenosis.

Additionally, irrespective of patients with AS, a REDUCE-IT clinical trial demonstrated that high-dose eicosapentaenoic acid (EPA) ethyl ester led to a 25% relative risk reduction in major cardiovascular events—encompassing cardiovascular death, nonfatal myocardial infarction, nonfatal stroke, coronary revascularization and hospitalization for unstable angina—compared with placebo [20].

In a sham-controlled study, rats underwent surgery to elevate cardiac afterload by inducing pressure overload, which led to cardiac hypertrophy. This pathomechanism is comparable to aortic valve stenosis-induced cardiac hypertrophy. Co-administration of DHA and flax lignan concentrate significantly reduced hemodynamic abnormalities, improved LV contractile function and attenuated both cardiomyocyte apoptosis and cellular oxidative stress. Furthermore, it markedly upregulated vascular endothelial growth factor (VEGF) expression and lowered Tumor Necrosis Factor-alpha (TNF-alpha) levels in serum [21]. Our observation of reduced DHA levels at six months post-TAVI may indicate a response to afterload reduction achieved through the enlargement of the aortic valve orifice area following TAVI. This could indicate the potentially beneficial impact of DHA supplementation for prevention of negative cardiac remodeling due to aortic valve stenosis.

AS-induced hypertrophy leads to higher uptake of free FAs with increased oxidation in cardiomyocytes [22]. In our study, elevated concentrations of C12 and C14—at six months post-procedure may suggest reduced uptake as a consequence of decreased afterload. The limitation of this hypothesis is the lack of concentrations of acyl-carnitine species or free carnitine.

Six months after the procedure, among the various MUFAs species, C14:1 was the sole acid to demonstrate a significant rise in levels relative to other time intervals. Overall MUFA concentrations remained unchanged when comparing pre-TAVI values to those six months post-intervention. Nevertheless, a significant uptick was evident at the six-month mark compared to measurements taken immediately following the procedure. A comparative study investigated the association between MUFA carbon chain length and CAD mortality. The results showed that long-chain MUFAs (LC-MUFAs) were inversely associated with CAD mortality, while very long-chain MUFAs (VLC-MUFAs) were directly associated with increased CAD mortality [23].

Long-chain SFAs were found to be associated with an elevated risk of CAD. Stearic acid directly contributes to dyslipidaemia through its potential to reduce HDL cholesterol levels. Moreover, palmitic acid has been associated with greater risks of AF, HF and mortality [24,25]. In our study total SFA and ECSFA levels remained largely unchanged between the pre-TAVI phase and six months post-procedure.

OCFAs demonstrate anti-inflammatory and antioxidant activities [26]. Studies have shown that higher dietary intake of OCFAs is correlated with a reduced risk of diabetes and CVD [27,28]. In our study no significant differences in total OCFA levels were observed between the pre-TAVI period and six months post-procedure. However, detailed analysis indicated a significant elevation in total OCFA levels at six months post-procedure compared to those recorded immediately after the procedure, with a particular increase noted in C17 concentrations (Supplementary Table S2). This may suggest a change in dietary habits and the overall diet of the patients after TAVI procedure.

We found also significant changes in C18, C28 and C25 FA concentrations immediately after TAVI and on the day following the procedure.

Although FA concentrations may change with fasting, we would like to highlight that all blood samples in our study were taken in a fasting state. Taking into account data showing serum FAs change after some anesthetic agents, including propofol, cyclopropane and nitrous oxide-halothane [29,30,31], we analyzed only patients undergoing transfemoral TAVI in local anesthesia. Our study was focused on long-term analysis after six months. Up to now there are no data indicating that transcatheter valve interventions may be associated with the change in serum FA concentrations. However, it is known that bariatric surgery decreases concentrations of seven non-esterified FAs (myristate, palmitoleate, palmitate, linoleate, oleate, stearate and arachidonate), as well as total medium-chain FA concentrations [32]. Our study revealed a significant elevation in total BCFA levels, including both iso (iso C16 and iso C17) and anteiso forms (anteiso C15 and anteiso C17). BCFAs are recognized for their ability to reduce the expression of genes involved in lipid synthesis and genes encoding pro-inflammatory proteins [33,34]. To date, no studies have assessed changes in BCFA levels in the context of aortic valve stenosis and its repair that indicate the beneficial effects of TAVI on FA profiles.

We found also increased lipid levels after TAVI. This could result from nonadherence to statin treatment and improved clinical condition of patients which could translate into a reduction of medications used.

To the best of our knowledge, this is the first study to comprehensively demonstrate significant changes in fatty acid profiles in patients with severe aortic stenosis undergoing transcatheter aortic valve implantation.

### Limitations of the Study

The follow-up period coincided with the COVID-19 pandemic. Due to related restrictions, elective hospital admissions and scheduled outpatient visits were suspended. As a result, the follow-up visit was limited to procedures directly related to the study. Consequently, standard laboratory tests (such as complete blood count, creatinine, CRP, etc.) could not be performed in the central laboratory. Therefore, only blood samples collected during the follow-up visit were analyzed for fatty acid profiles within the framework of this study. This explains the absence of standard laboratory data at the follow-up time point.

Due to the COVID pandemic, the advanced age of the study population (81 ± 5.6 years) and the high prevalence of cardiovascular comorbidities, consistent adherence to prescribed medications was not achieved. Moreover, as patients experienced improved well-being and functional status after the procedure, some discontinued their prescribed medications, including statins, as these drugs are not routinely prescribed after TAVI. Additionally, many patients lived a considerable distance from our center, which created transportation difficulties during the COVID pandemic and led to refusal or inability to attend the 6-month follow-up visit.

## 5. Conclusions

In conclusion, our study demonstrated significant changes in lipid and FA profiles following TAVI, with distinct patterns observed in different FA subgroups. This is the first study to comprehensively investigate these metabolic shifts in the context of TAVI. Our study findings underscore the complex interplay between aortic valve parameters and FA concentrations. Our findings provide valuable insights into this evolving area of research and establish a basis for future investigations to confirm and expand upon these results.

## Figures and Tables

**Table 1:** Clinical characteristics of the study group.

Medical Condition	Number (Male/Female)
Hypertension	23 (10/13), %
Hypercholesterolemia	24 (11/13)
Diabetes type 2	6 (2/4)
Smoking	3 (2/1)
Heart failure	23 (12/11)
Coronary artery disease	14 (9/5)
Percutaneous coronary intervention	6 (3/3)
Myocardial infarction	5 (3/2)
Chronic kidney disease	14 (7/7)
Chronic obstructive pulmonary disease	6 (3/3)
Previous open-heart surgery	3 (2/1)
Previous coronary artery bypass graft surgery	3 (2/1)
Previous valve surgery	0 (0/0)
Other valve disease (mitral and/or aortic regurgitation)	8 (2/6)
Peripheral vascular disease	4 (3/1)
Carotid stenosis > 50%	1 (0/1)
Stroke and/or transient ischemic attack	2 (1/1)
Atrial fibrillation	8 (3/5)
Previous implantation of pacemaker	2 (0/2)
Porcelain aorta	0 (0/0)
Neoplasmatic disease	2 (0/2)

**Table 2:** Selected biochemical and anthropometric characteristics in the study group.

	Baseline	Reference Value
Sex (M/F)	12/13	-
Age (year)	81 ± 5.6	-
BMI (kg/m^2^)	30 ± 4.4	18.5–24.9
Creatinine (mg/dL)	1.15 ± 0.4	0.6–1.3
eGFR-MDRD (mL/min/1.73 m^2^)	43.5 ± 9	120–130
Calcium score of aortic valve	2766 ± 1899	M < 1600; F < 800
AV Max Pressure Gradient (mmHg)	75.5 ± 20.1	<16
AV Mean Pressure Gradient (mmHg)	45.5 ± 14.4	<10
AVA (cm^2^)	0.8 ± 0.1	2.5–3.5
LVEF (%)	55 ± 14.3	M: 52–72; F: 54–74
Glycemia (mg/dL)	102 ± 38.5	70–99
Hematocrit (%)	38 ± 3.3	36–45
Platelets (×10^9^/L)	191 ± 43.5	150–400
Leucocytes (×10^9^/L)	6.7 ± 1.8	4–10

Values are mean ± standard deviation (SD). BMI—body mass index, eGFR-MDRD—estimated glomerular filtration rate-Modification of Diet in Renal Disease, AVA—aortic valve area, LVEF—left ventricular ejection fraction.

**Table 3:** Lipid parameters before TAVI and six months after procedure.

Lipid Parameter [mg/ds]	Before TAVI	After 6 Months	*p* Value
TC	149 ± 42.7	179 ± 39.7	0.001
TG	96.6 ± 34.1	142 ± 49.4	<0.001
HDL	37.0 ± 9.28	40.8 ± 7.60	0.005
LDL	91.8 ± 33.8	118 ± 43.8	0.010

Values are mean ± standard deviation (SD). TC—total cholesterol, TG—triglycerides, HDL—high-density lipoprotein cholesterol, LDL—low-density lipoprotein cholesterol.

## Data Availability

The data presented in this study are available on request from the corresponding author. The data are not publicly available due to privacy.

## Supplementary Materials

The following supporting information can be downloaded at: https://www.mdpi.com/article/10.3390/metabo15120774/s1. Table S1: Effect of TAVI procedure on fatty acids concentration (µM) in the serum of patients; Table S2: Effect of TAVI procedure and surgical intervention on fatty acids concentration (µM) in the serum of patients.

## References

[1] Badiani S., van Zalen J., Treibel T.A., Bhattacharyya S., Moon J.C., Lloyd G. (2016). Aortic Stenosis, a Left Ventricular Disease: Insights from Advanced Imaging. Curr. Cardiol. Rep..

[2] Miller J.D., Weiss R.M., Heistad D.D. (2011). Calcific aortic valve stenosis: Methods, models, and mechanisms. Circ. Res..

[3] Otto C.M., Kuusisto J., Reichenbach D.D., Gown A.M., O’Brien K.D. (1994). Characterization of the Early Lesion of ‘Degenerative’ Valvular Aortic Stenosis Histological and Immunohistochemical Studies. Circulation.

[4] Rajamannan N.M., Evans F.J., Aikawa E., Grande-Allen K.J., Demer L.L., Heistad D.D., Simmons C.A., Masters K.S., Mathieu P., O’Brien K.D. (2011). Calcific aortic valve disease: Not simply a degenerative process: A review and agenda for research from the national heart and lung and blood institute aortic stenosis working group. Circulation.

[5] Maki K.C., Dicklin M.R., Kirkpatrick C.F. (2021). Saturated fats and cardiovascular health: Current evidence and controversies. J. Clin. Lipidol..

[6] Tan T., Luo Y., Sun W., Li X. (2023). Effects of Branched-Chain Fatty Acids Derived from Yak Ghee on Lipid Metabolism and the Gut Microbiota in Normal-Fat Diet-Fed Mice. Molecules.

[7] Wang H., Steffen L.M., Vessby B., Basu S., Steinberger J., Moran A., Jacobs D.R., Hong C.-P., Sinaiko A.R. (2011). Obesity Modifies the Relations Between Serum Markers of Dairy Fats and Inflammation and Oxidative Stress Among Adolescents. Obesity.

[8] Bäck M. (2021). Fatty acids and aortic valve stenosis. Kardiol. Pol..

[9] Mora S., Dugani S.B., Moorthy M.V., Li C., Demler O.V., Alsheikh-Ali A.A., Ridker P.M., Glynn R.J., Mora S. (2021). Association of Lipid, Inflammatory, and Metabolic Biomarkers With Age at Onset for Incident Coronary Heart Disease in Women. JAMA Cardiol..

[10] Artiach G., Bäck M. (2020). Omega-3 Polyunsaturated Fatty Acids and the Resolution of Inflammation: Novel Therapeutic Opportunities for Aortic Valve Stenosis?. Front. Cell Dev. Biol..

[11] Artiach G., Carracedo M., Plunde O., Wheelock C.E., Thul S., Sjövall P., Franco-Cereceda A., Laguna-Fernandez A., Arnardottir H., Bäck M. (2020). Omega-3 Polyunsaturated Fatty Acids Decrease Aortic Valve Disease Through the Resolvin E1 and ChemR23 Axis. Circulation.

[12] Joseph J., Naqvi S.Y., Giri J., Goldberg S. (2017). Aortic Stenosis: Pathophysiology, Diagnosis, and Therapy. Am. J. Med..

[13] Zheng K.H., Tzolos E., Dweck M.R. (2020). Pathophysiology of Aortic Stenosis and Future Perspectives for Medical Therapy. Cardiol. Clin..

[14] Takagi H., Hari Y., Nakashima K., Kuno T., Ando T. (2020). Echocardiographic outcomes from seven randomized trials of transcatheter versus surgical aortic valve replacement. J. Cardiovasc. Med..

[15] Adams D.H., Popma J.J., Reardon M.J., Yakubov S.J., Coselli J.S., Deeb G.M., Gleason T.G., Buchbinder M., Hermiller J., Kleiman N.S. (2014). Transcatheter Aortic-Valve Replacement with a Self-Expanding Prosthesis. N. Engl. J. Med..

[16] Gleason T.G., Reardon M.J., Popma J.J., Deeb G.M., Yakubov S.J., Lee J.S., Kleiman N.S., Chetcuti S., Hermiller J.B., Heiser J. (2018). 5-Year Outcomes of Self-Expanding Transcatheter Versus Surgical Aortic Valve Replacement in High-Risk Patients. J. Am. Coll. Cardiol..

[17] Vahanian A., Beyersdorf F., Praz F., Milojevic M., Baldus S., Bauersachs J., Capodanno D., Conradi L., De Bonis M., De Paulis R. (2022). 2021 ESC/EACTS Guidelines for the management of valvular heart disease: Developed by the Task Force for the management of valvular heart disease of the European Society of Cardiology (ESC) and the European Association for Cardio-Thoracic Surgery (EACTS). Eur. Heart J..

[18] Zornitzki L., Ben-Shoshan J. (2025). Predictors of long-term survival in patients undergoing TAVR: Recent advances. A narrative review. Pol. Heart J. (Kardiol. Pol.).

[19] Xiong T.Y., Liu C., Liao Y.B., Zheng W., Li Y.J., Li X., Ou Y., Wang Z.J., Wang X., Li C.M. (2020). Differences in metabolic profiles between bicuspid and tricuspid aortic stenosis in the setting of transcatheter aortic valve replacement. BMC Cardiovasc. Disord..

[20] Bhatt D.L., Steg P.G., Miller M., Brinton E.A., Jacobson T.A., Ketchum S.B., Doyle R.T., Juliano R.A., Jiao L., Granowitz C. (2019). Cardiovascular Risk Reduction with Icosapent Ethyl for Hypertriglyceridemia. N. Engl. J. Med..

[21] Ghule A.E., Kandhare A.D., Jadhav S.S., Zanwar A.A., Bodhankar S.L. (2015). Omega-3-fatty acid adds to the protective effect of flax lignan concentrate in pressure overload-induced myocardial hypertrophy in rats via modulation of oxidative stress and apoptosis. Int. Immunopharmacol..

[22] Voros G., Ector J., Garweg C., Droogne W., Van Cleemput J., Peersman N., Vermeersch P., Janssens S. (2018). Increased Cardiac Uptake of Ketone Bodies and Free Fatty Acids in Human Heart Failure and Hypertrophic Left Ventricular Remodeling. Circ. Heart Fail..

[23] Li Z., Zhang Y., Su D., Lv X., Wang M., Ding D., Ma J., Xia M., Wang D., Yang Y. (2014). The opposite associations of long-chain versus very long-chain monounsaturated fatty acids with mortality among patients with coronary artery disease. Heart.

[24] Hu F.B., Stampfer M.J., Manson J.A.E., Ascherio A., Colditz G.A., Speizer F.E., Hennekens C.H., Willett W.C. (1999). Dietary saturated fats and their food sources in relation to the risk of coronary heart disease in women. Am. J. Clin. Nutr..

[25] Lemaitre R.N., King I.B. (2022). Very long-chain saturated fatty acids and diabetes and cardiovascular disease. Curr. Opin. Lipidol..

[26] Řezanka T., Sigler K. (2009). Odd-numbered very-long-chain fatty acids from the microbial, animal and plant kingdoms. Prog. Lipid Res..

[27] Warensjö E., Jansson J.-H., Berglund L., Boman K., Ahrén B., Weinehall L., Lindahl B., Hallmans G., Vessby B. (2004). Estimated intake of milk fat is negatively associated with cardiovascular risk factors and does not increase the risk of a first acute myocardial infarction. A prospective case-control study. Br. J. Nutr..

[28] Krachler B., Norberg M., Eriksson J.W., Hallmans G., Johansson I., Vessby B., Weinehall L., Lindahl B. (2008). Fatty acid profile of the erythrocyte membrane preceding development of Type 2 diabetes mellitus. Nutr. Metab. Cardiovasc. Dis..

[29] Nummela A.J., Laaksonen L.T., Laitio T.T., Kallionpää R.E., Långsjö J.W., Scheinin J.M., Vahlberg T.J., Koskela H.T., Aittomäki V., Valli K.J. (2022). Effects of dexmedetomidine, propofol, sevoflurane and S-ketamine on the human metabolome: A randomised trial using nuclear magnetic resonance spectroscopy. Eur. J. Anaesthesiol..

[30] Feistritzer H.J., Kurz T., Vonthein R., Schröder L., Stachel G., Eitel I., Marquetand C., Saraei R., Kirchhof E., Heringlake M. (2025). Effect of Valve Type and Anesthesia Strategy for TAVR: 5-Year Results of the SOLVE-TAVI Trial. J. Am. Coll. Cardiol..

[31] Cooperman L.H. (1970). PLASMA FREE FATTY ACID LEVELS DURING GENERAL ANAESTHESIA AND OPERATION IN MAN. Br. J. Anaesth..

[32] Sharma C., Platat C., Gariballa S., Al Muhairi S.J., Al Aidaros A., Mannaerts G.H.H., Al Afari H.S., Yasin J., Al-Dirbashi O.Y., Alkaabi J. (2021). Metabolomic Profiling of Lipids and Fatty Acids: 3 Years Postoperative Laparoscopic Sleeve Gastrectomy. Biology.

[33] Mika A., Stepnowski P., Kaska L., Proczko M., Wisniewski P., Sledzinski M., Sledzinski T. (2016). A comprehensive study of serum odd- and branched-chain fatty acids in patients with excess weight. Obesity.

[34] Czumaj A., Śledziński T., Mika A. (2022). Branched-Chain Fatty Acids Alter the Expression of Genes Responsible for Lipid Synthesis and Inflammation in Human Adipose Cells. Nutrients.

