# Supplementary material for: Evaluation of the Effect of Transcatheter Aortic Valve Implantation in Patients with Severe Aortic Stenosis on the Concentration of the Fatty Acids Involved in Inflammation
Source: Metabolites. 2025 Nov 29;15(12):774. doi: 10.3390/metabo15120774 (PMC12734766; doi:10.3390/metabo15120774)
Supplement: Supplementary file 1 [file metabolites-15-00774-s001.zip › Table S1-1.pdf]

Table S1. Effect of TAVI procedure on fatty acids concentration (μM) in the serum of patients.

			n=25
Fatty acids	Before TAVI	6m after TAVI	p-value
ALA	19.9 ± 6.40	32.8 ± 12.3	0.003
ETA	7.64 ± 3.11	8.12 ± 3.63	NS
EPA	68.2 ± 32.9	73.7 ± 27.6	NS
DPA n3	32.0 ± 8.85	31.3 ± 10.4	NS
DHA	141 ± 53.0	117 ± 48.0	0.001
Total n3 PUFA	284 ± 112	265 ± 105	0.039
LA	1645 ± 595	1657 ± 603	NS
EDA	13.1 ± 5.14	13.4 ± 5.25	NS
DGLA	105 ± 36.0	112 ± 39.7	NS
ARA	515 ± 154	462 ± 119	0.015
AdA	12.3 ± 4.23	11.6 ± 4.48	0.050
DPA n6	6.83 ± 2.05	6.82 ± 3.05	NS
Total n6 PUFA	2252 ± 728	2216 ± 692	NS
iso C14	0.91 ± 0.21	1.05 ± 0.39	NS
iso C15	1.82 ± 0.74	2.31 ± 1.10	NS
iso C16	4.51 ± 1.46	5.66 ± 2.17	0.001
iso C17	8.19 ± 2.92	9.13 ± 3.81	0.045
Total iso BCFA	17.3 ± 6.58	19.2 ± 7.85	0.001
anteiso C15	3.02 ± 1.14	4.31 ± 2.06	0.044
anteiso C17	7.07 ± 3.27	10.2 ± 4.11	0.001
Total anteiso BCFA	10.3 ± 3.96	14.3 ± 5.49	0.001
Total BCFA	31.0 ± 15.3	34.6 ± 14.8	0.013
C12	1.84 ± 0.89	2.45 ± 1.23	0.031
C14	59.7 ± 19.7	83.7 ± 37.2	0.039
C16	1671 ± 526	1733 ± 606	NS
C18	523 ± 147	566 ± 172	NS
C20	10.8 ± 2.87	10.1 ± 3.04	NS
C22	18.5 ± 4.66	18.5 ± 4.98	NS
C24	17.9 ± 5.01	17.1 ± 4.83	NS
C26	0.72 ± 0.27	0.63 ± 0.16	NS
Total ECSFA	2082 ± 439	2349 ± 793	NS
C13	0.96 ± 0.28	1.09 ± 0.50	NS
C15	24.5 ± 6.42	27.7 ± 11.2	NS
C17	27.8 ± 8.04	28.7 ± 10.3	NS
C19	2.30 ± 0.97	2.10 ± 0.71	NS
C21	1.60 ± 0.49	1.66 ± 0.61	NS
C23	8.25 ± 2.47	7.67 ± 2.02	NS
C25	0.69 ± 0.26	0.63 ± 0.18	NS
Total OCFA	57.2 ± 12.9	61.3 ± 21.6	NS
Total SFA	2308 ± 578	2464 ± 756	NS
C14:1	2.82 ± 1.23	4.48 ± 2.39	0.013
C16:1	297 ± 85.4	312 ± 114	NS
C18:1	1854 ± 740	1972 ± 772	NS
C20:1	13.6 ± 6.73	12.0 ± 4.71	NS
C22:1	1.36 ± 0.51	1.07 ± 0.46	NS
Total MUFA	2158 ± 830	2314 ± 916	NS

Values as mean ± SD. AdA – adrenic acid, ALA – alfa linolenic acid, ARA_ arachidonic acid, BCFA – branched chain FA, DHA – docosahexaenoic acid, DPA – docosapentaenoic acid, ECFA – even chain FA, EDA – eicosadienoic acid, EPA – eicosapentaenoic acid, ETA – eicosatetraenoic acid, MUFA – monounsaturated FA, OCFA – odd chain FA, PUFA – polyunsaturated FA, SFA- saturated FA. NS—nonsignificant. SFA is a sum of ECSFA, OCFA and BCFA.
