# Supplementary material for: Evaluation of the Effect of Transcatheter Aortic Valve Implantation in Patients with Severe Aortic Stenosis on the Concentration of the Fatty Acids Involved in Inflammation
Source: Metabolites. 2025 Nov 29;15(12):774. doi: 10.3390/metabo15120774 (PMC12734766; doi:10.3390/metabo15120774)
Supplement: Supplementary file 1 [file metabolites-15-00774-s001.zip › Table S2-1.pdf]

Table S2. Effect of TAVI procedure and surgical intervention on fatty acids concentration (μM) in the serum of patients.

Fatty acids	**TAVI - Transcatheter Aortic Valve Implantation**				**p-value**				
	Before	After	1d after	6m after	6m vs. before	6m vs. after	6m vs. 1d after	after vs. before	1d a vs. before
ALA	19.9 ± 6.40	18.5 ± 6.02	15.0 ± 4.98	32.8 ± 12.3	<0.001	<0.001	<0.001	NS	NS
ETA	7.64 ± 3.11	6.04 ± 2.06	7.25 ± 2.06	8.12 ± 3.63	NS	0.029	NS	NS	NS
EPA	68.2 ± 32.9	60.7 ± 23.4	53.7 ± 21.0	73.7 ± 27.6	NS	NS	0.021	NS	NS
DPA n3	32.0 ± 8.85	23.41 ± 5.59	29.0 ± 5.74	31.3 ± 10.4	NS	0.014	NS	0.007	NS
DHA	141 ± 53.0	115 ± 38.6	118 ± 38.9	117 ± 48.0	NS	NS	NS	NS	NS
Total n3 PUFA	284 ± 112	225 ± 55.7	229 ± 68.4	265 ± 105	NS	NS	NS	NS	NS
LA	1645 ± 595	1303 ± 365	1481 ± 450	1657 ± 603	NS	NS	NS	NS	NS
EDA	13.1 ± 5.14	9.89 ± 3.90	9.32 ± 2.68	13.4 ± 5.25	NS	0.037	0.005	NS	0.011
DGLA	105 ± 36.0	83.3 ± 16.0	83.6 ± 22.3	112 ± 39.7	NS	0.030	0.037	NS	NS
ARA	515 ± 154	411 ± 76.9	461 ± 114	462 ± 119	NS	NS	NS	NS	NS
AdA	12.3 ± 4.23	10.2 ± 2.36	10.9 ± 2.58	11.6 ± 4.48	NS	NS	NS	NS	NS
DPA n6	6.83 ± 2.05	6.83 ± 3.09	6.31 ± 2.01	6.82 ± 3.05	NS	NS	NS	NS	NS
Total n6 PUFA	2252 ± 728	1879 ± 472	2060 ± 516	2216 ± 692	NS	NS	NS	NS	NS
iso C14	0.91 ± 0.21	0.79 ± 0.20	0.93 ± 0.33	1.05 ± 0.39	NS	0.021	NS	NS	NS
iso C15	1.82 ± 0.74	1.61 ± 0.50	1.34 ± 0.45	2.31 ± 1.10	NS	NS	0.003	NS	NS
iso C16	4.51 ± 1.46	3.89 ± 1.05	4.01 ± 0.95	5.66 ± 2.17	NS	0.004	0.011	NS	NS
iso C17	8.19 ± 2.92	8.56 ± 2.99	8.12 ± 2.52	9.13 ± 3.81	NS	NS	NS	NS	NS
Total iso BCFA	17.3 ± 6.58	15.2 ± 4.36	14.2 ± 3.14	19.2 ± 7.85	NS	NS	NS	NS	NS
anteiso C15	3.02 ± 1.14	3.20 ± 1.01	3.02 ± 1.15	4.31 ± 2.06	NS	NS	0.039	NS	NS
anteiso C17	7.07 ± 3.27	7.97 ± 3.12	7.33 ± 2.61	10.2 ± 4.11	0.020	NS	0.039	NS	NS
Total anteiso BCFA	10.3 ± 3.96	11.4 ± 4.24	10.3 ± 3.34	14.3 ± 5.49	0.025	NS	0.034	NS	NS
Total BCFA	31.0 ± 15.3	27.5 ± 9.16	25.3 ± 7.33	34.6 ± 14.8	NS	NS	NS	NS	NS
C12	1.84 ± 0.89	1.89 ± 0.93	2.06 ± 0.91	2.45 ± 1.23	NS	NS	NS	NS	NS
C14	59.7 ± 19.7	51.5 ± 11.8	54.1 ± 19.1	83.7 ± 37.2	0.021	<0.001	0.002	NS	NS
C16	1671 ± 526	1308 ± 334	1632 ± 419	1733 ± 606	NS	0.034	NS	NS	NS
C18	523 ± 147	416 ± 79.1	437 ± 79.8	566 ± 172	NS	0.002	0.021	0.022	NS
C20	10.8 ± 2.87	9.46 ± 1.50	9.41 ± 1.57	10.1 ± 3.04	NS	NS	NS	NS	NS
C22	18.5 ± 4.66	16.6 ± 3.06	17.1 ± 2.88	18.5 ± 4.98	NS	NS	NS	NS	NS
C24	17.9 ± 5.01	16.1 ± 3.72	16.5 ± 4.19	17.1 ± 4.83	NS	NS	NS	NS	NS
C26	0.72 ± 0.27	0.91 ± 0.35	0.99 ± 0.26	0.63 ± 0.16	NS	0.031	0.002	NS	0.021

Total ECSFA	2082 ± 439	1793 ± 413	2150 ± 522	2349 ± 793	NS	0.016	NS	NS	NS
C13	0.96 ± 0.28	0.87 ± 0.22	0.92 ± 0.22	1.09 ± 0.50	NS	NS	NS	NS	NS
C15	24.5 ± 6.42	21.9 ± 4.80	22.9 ± 8.22	27.7 ± 11.2	NS	NS	NS	NS	NS
C17	27.8 ± 8.04	23.2 ± 6.72	23.6 ± 5.06	28.7 ± 10.3	NS	0.041	NS	NS	NS
C19	2.30 ± 0.97	1.93 ± 0.55	1.85 ± 0.62	2.10 ± 0.71	NS	NS	NS	NS	NS
C21	1.60 ± 0.49	1.62 ± 0.55	1.59 ± 0.58	1.66 ± 0.61	NS	NS	NS	NS	NS
C23	8.25 ± 2.47	7.23 ± 1.61	7.22 ± 1.53	7.67 ± 2.02	NS	NS	NS	NS	NS
C25	0.69 ± 0.26	0.65 ± 0.19	0.92 ± 0.28	0.63 ± 0.18	NS	NS	0.005	NS	0.044
Total OCFA	57.2 ± 12.9	47.0 ± 8.21	52.6 ± 11.1	61.3 ± 21.6	NS	0.035	NS	NS	NS
Total SFA	2308 ± 578	1915 ± 433	2270 ± 534	2464 ± 756	NS	0.027	NS	NS	NS
C14:1	2.82 ± 1.23	2.08 ± 0.89	2.21 ± 0.90	4.48 ± 2.39	0.005	<0.001	<0.001	NS	NS
C16:1	297 ± 85.4	253 ± 84.7	258.9 ±70.1	312 ± 114	NS	NS	NS	NS	NS
C18:1	1854 ± 740	1484 ± 425	1913 ± 553	1972 ± 772	NS	0.048	NS	NS	NS
C20:1	13.6 ± 6.73	11.1 ± 5.44	12.1 ± 3.64	12.0 ± 4.71	NS	NS	NS	NS	NS
C22:1	1.36 ± 0.51	1.10 ± 0.46	1.19 ± 0.43	1.07 ± 0.46	NS	NS	NS	NS	NS
Total MUFA	2158 ± 830	1755 ± 489	2200 ± 631	2314 ± 916	NS	0.047	NS	NS	NS

Values are mean ± SD; value of $p < 0.05$ was considered statistically significant. For quantitative measures, the ANOVA test with Bonferroni correction were used. AdA – adrenic acid, ALA – alfa linolenic acid, ARA_ arachidonic acid, BCFA – branched chain FA, DHA – docosahexaenoic acid, DPA – docosapentaenoic acid, ECFA – even chain FA, EDA – eicosadienoic acid, EPA – eicosapentaenoic acid, ETA – eicosatetraenoic acid, MUFA – monounsaturated FA, OCFA – odd chain FA, PUFA – polyunsaturated FA, SFA- saturated FA. NS—nonsignificant. SFA is a sum of ECSFA, OCFA and BCFA.
